# Brain (re)organisation following amputation: Implications for phantom limb pain

**DOI:** 10.1016/j.neuroimage.2020.116943

**Published:** 2020-09

**Authors:** Tamar R. Makin, Herta Flor

**Affiliations:** a Institute of Cognitive Neuroscience, University College London, London, United Kingdom; b Wellcome Centre for Human Neuroimaging, University College London, London, UK; c Institute of Cognitive and Clinical Neuroscience, Central Institute of Mental Health, Medical Faculty Mannheim, Heidelberg University, Mannheim, Germany; d Department of Psychology, School of Social Sciences, University of Mannheim, Germany; e Center for Neuroplasticity and Pain (CNAP), Department of Health Science and Technology, Aalborg University, Aalborg, Denmark

**Keywords:** Neuroimaging, Cortical reorganisation, Phantom limb pain, Preserved function, Multivariate analysis, Pain treatment, Use-dependent plasticity

## Abstract

Following arm amputation the region that represented the missing hand in primary somatosensory cortex (S1) becomes deprived of its primary input, resulting in changed boundaries of the S1 body map. This remapping process has been termed ‘reorganisation’ and has been attributed to multiple mechanisms, including increased expression of previously masked inputs. In a maladaptive plasticity model, such reorganisation has been associated with phantom limb pain (PLP). Brain activity associated with phantom hand movements is also correlated with PLP, suggesting that preserved limb functional representation may serve as a complementary process. Here we review some of the most recent evidence for the potential drivers and consequences of brain (re)organisation following amputation, based on human neuroimaging. We emphasise other perceptual and behavioural factors consequential to arm amputation, such as non-painful phantom sensations, perceived limb ownership, intact hand compensatory behaviour or prosthesis use, which have also been related to both cortical changes and PLP. We also discuss new findings based on interventions designed to alter the brain representation of the phantom limb, including augmented/virtual reality applications and brain computer interfaces. These studies point to a close interaction of sensory changes and alterations in brain regions involved in body representation, pain processing and motor control. Finally, we review recent evidence based on methodological advances such as high field neuroimaging and multivariate techniques that provide new opportunities to interrogate somatosensory representations in the missing hand cortical territory. Collectively, this research highlights the need to consider potential contributions of additional brain mechanisms, beyond S1 remapping, and the dynamic interplay of contextual factors with brain changes for understanding and alleviating PLP.

## Highlights

• Technological advancements provide new insight into the neural basis of phantom pain.
• Traditional mechanistic accounts of remapping in somatosensory cortex are incomplete.
• Related contextual factors such as adaptive behaviour will contribute to brain plasticity.
• A broader mechanistic focus beyond primary sensorimotor cortex is needed.
• Plasticity and stability of the sensorimotor body maps may vary across time scales.

## 1 Introduction

Three decades ago it was first demonstrated that the sensory and motor maps in the adult primate brain can change as a consequence of injury as well as in response to training and stimulation (Jenkins et al., 1990; Kaas et al., 1990; Merzenich et al., 1983; Rajan et al., 1993; Sanes et al., 1988), and that these organisational changes are not limited to early brain development. This remapping has been attributed to an unmasking of normally inhibited connections between representational areas (Harding-Forrester and Feldman, 2018; Li et al., 2014), changes in subcortical projections to cortex (Jain et al., 2008) and even structural changes such as axonal sprouting (Florence et al., 1998; Jones and Pons, 1998) (though see (Chand and Jain, 2015) on changes after spinal cord injury). Subsequently, alterations in the organisation of sensory maps have been associated with a number of perceptual and behavioural changes, with many of them viewed as maladaptive, ranging from tinnitus to focal dystonia and phantom limb pain (PLP). However, these map changes have also been related to adaptive behaviors, such as improved sensory discrimination, advanced musical training and recovery from stroke (Flor and Diers, 2009). Here we review recent evidence on map changes in the primary somatosensory cortex (S1), and its association to PLP, based on recent neuroimaging studies in humans.

## *2* The sensorimotor homunculus – a neuroimaging perspective

Topographic body representation is one of the foundational organising principles in the brain. Multiple reports from the late 19th century observed that localised electric stimulation in animals evokes individualised movements of specific body parts (Ferrier, 1873). This work was refined by Penfield and colleagues, who reported a body-part map (somatotopy) along the human primary motor cortex (M1) (Penfield and Boldrey, 1937). Benefiting from verbal reports of their awake patients, Penfield and colleagues were able to infer that a second body map, relating to sensory perception, existed adjacently in the postcentral gyrus. These classical results, later elaborated in both animal (Romo et al., 1998, 2000; Tabot et al., 2013) and human cortical stimulation studies (Flesher et al., 2016; Hughes et al., 2020), established the role of S1 in eliciting key aspects of sensory bodily perception (e.g. stimulus modality, location, frequency and amplitude; note that S1 stimulation did not elicit painful sensations). More recent work, specifically focused on the causal role of S1, indicates that somatosensory perception might not be as heavily reliant on its processing (Medina and Rapp, 2014). Instead, S1 functioning may be particularly important for the consolidation of sensory and motor learning (Kumar et al., 2019; Mathis et al., 2017; Hong et al., 2018).

The consistent organisation of the sensorimotor maps was summarized in the canonical illustration of the human homunculus by Penfield and colleagues. However, despite this simplified illustration, the authors emphasised that the body map contains fuzzy boundaries, due to overlapping representations across body parts (Catani, 2017). Further work over the course of the 20th century revealed that although the motor map is best defined as crudely organised (Schieber, 2001), S1 contains detailed representations of specific body parts characterised by clearer boundaries (Kaas et al., 1979). The most striking, fine-grained organisation exists for the hand, where each of the digits and digit pads are represented separately and adjacently (Merzenich et al., 1983a), resulting in a detailed hand map.

**Fig. 1:**
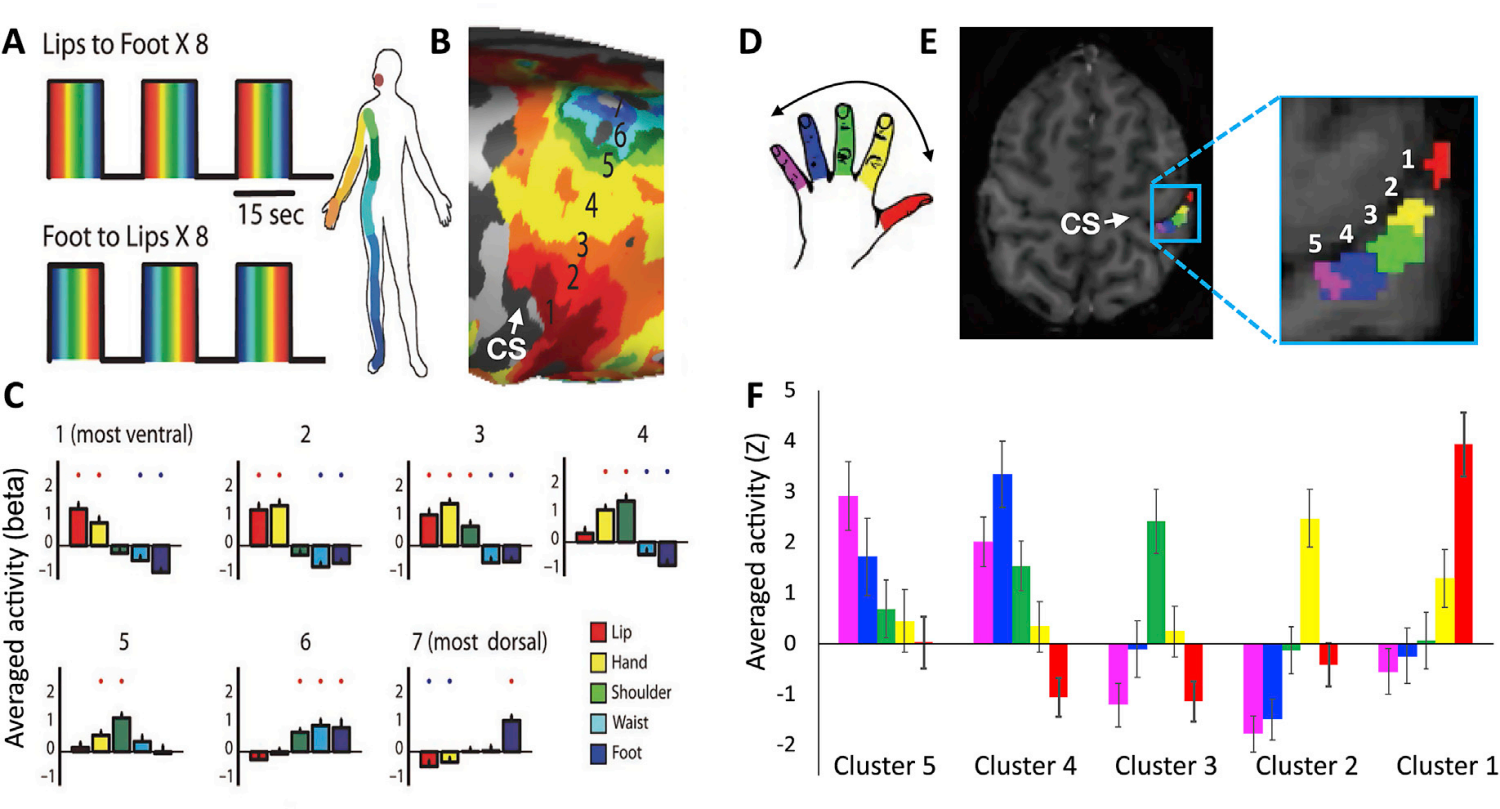
Somatotopic mapping of the entire body (A–C) and of the hand (D–E) in primary somatosensory cortex (S1) as revelaed by human task-based fMRI. Somatotopies are often studied using a travelling wave (also known as phase encoding) experimental design, where each of the body parts is stimulated sequentially in a set cycle (A,D). This techinque is designed to identify brain areas showing body part selectivity, such that each colour in the maps indicates selectivity to one body part in the sequence over all others (B – group map, E − sample participant). A further characterising feature of somatotopic representation is a gradient in selectivity, such that neighbouring body parts show greater overlap in cortical activity. This gradient can be observed using block- or event-related designs, where activity for each of the body parts can be assessed independently (C,F). Note that to avoid circular analysis, the activity gradients shown in (C,F) were extracted from independent regions of interest, based on the maps shown in (A,D). A-C was adapted from (Tal et al., 2017); D-E was adapted from Sanders et al. (2019).

The rise of neuroimaging allowed the investigation of somatotopic maps in the healthy human brain using PET (Fox et al., 1987), MEG (Nakamura et al., 1998), task-based fMRI (Glasser et al., 2016), resting-state functional connectivity (Yeo et al., 2011) and even structural neuroimaging techniques (e.g. DTI tractography (Behrens et al., 2003)). Unlike direct brain stimulation and recordings, human neuroimaging provided opportunities to identify body and hand representation beyond S1/M1 – namely in the cerebellum (Hahamy and Makin, 2019; Yeo et al., 2011), basal ganglia (Zeharia et al., 2015), operculum and insula (Brooks et al., 2005), supplementary motor cortex (Zeharia et al., 2012), occipitotemporal cortex (Orlov et al., 2010), parietal cortex (Huang et al., 2012; Zeharia et al., 2019) and more. Despite these findings indicating that body representation should not be studied in S1 in isolation (Longo et al., 2010), in the rest of this paper we will primarily focus on S1 body representation as identified with task-based fMRI (see Fig. 1 for an illustrating example). We will not consider the literature on M1 (re)mapping nor will we discuss the interactions of S1 changes with peripheral factors, although we acknowledge their importance in understanding S1 organisation and PLP.

There are several methodological considerations that have constrained the study of S1 somatotopy, which should be taken into consideration before interpreting this wealth of literature. First, due to the need for multiple repetitions of each condition, and the limited time-frame of a typical neuroimaging session, very few studies attempted to reconstruct the full homunculus. With few exceptions (e.g. (Saadon-Grosman et al., 2015; Tal et al., 2017; Zeharia et al., 2019, 2015; 2012)), most studies focused on the relative layout of several distinct body parts, and most prominently the foot, hand and mouth. This leaves potential gaps in the layout of the human somatotopy (e.g., it is still debated whether the hand representation neighbours the upper (Moulton et al., 2009) or lower (Kuehn et al., 2017) face representation). Other studies have focused on detailed investigations of one body part (most commonly the hand). Here the classical studies were restricted by limited resolution, making the dissociation of individual digits relatively noisy (Overduin and Servos, 2004). Recent advances in fMRI, and in particular techniques that provide increased signal to noise ratio and spatial resolution (multiband sequences and 7T MRI), offer new opportunities to overcome some of these technical issues. These new advancements allow for characterisation of the S1 hand maps with unprecedented detail: 7T fMRI permits identification of individual-digit finger maps with high intra- and inter-subject consistency (Kolasinski et al., 2016; Sanchez-Panchuelo et al., 2010). Structural techniques provide a first glimpse into the anatomical constraints of this organisation, for example, the hand-face border (Kuehn et al., 2017).

A further constraint for S1 mapping relates to the means of stimulation available, particularly when considering the restricted environment of MRI scanners, safety considerations, limited physical space and magnetic field distortions. Additionally, the peripheral nervous system has been shown to display high adaptation rates to repeated tactile stimuli (O’Mara et al., 1988). For these reasons, many researchers opted to using active paradigms, where participants are cued to move different body parts resulting in robust S1 activity (Makin et al., 2013a; Zeharia et al., 2015). However, this approach raises its own confounds, in particular unreliable delineation of S1 from M1 with standard acquisition resolution and pre-processing techniques. Moreover, movement-related S1 activity differs from passive sensory stimulation S1 activity, in that it recruits multiple additional inputs, including efferent signals from the motor system (London and Miller, 2013), deep cutaneous and proprioceptive peripheral signals (Grigg, 1994) and even cognitive or multisensory top-down modulatory inputs (Kuehn et al., 2018; Puckett et al., 2017; Shokur et al., 2013), though see (Berlot et al., 2019) and (Sanders et al., 2019) for similarities across multiple representational features of the hand and (Striem-Amit et al., 2018) for similarities in homunculus remapping between active and passive paradigms.

Non-invasive research in humans has also opened up new opportunities to study the role of S1 in pain processing, and specifically what are the organising principles of nociception in S1. Combined with electrophysiological research in both animals and humans, the evidence is inconclusive. While some studies demonstrated that painful stimuli are being processed in S1 (Tommerdahl et al., 1998; Vierck et al., 2013), other studies suggested that the observed activity does not reflect the nociceptive input per se, but rather other aspects relating to pain such as attention, salience or expectation (Bushnell et al., 1999; Mouraux and Iannetti, 2009). The spatiotemporal attributes of pain versus touch representation in S1 have also been debated, with some studies showing overlap in topographic finger representation (Mancini et al., 2012), while others identified differential organisation (Ploner et al., 2000). In the context of our review, it is important to note that nerve injury such as following amputation is known to trigger central sensitisation, whereby painful stimulation can activate S1 indirectly, due to plasticity in the dorsal pathway in the spinal cord (Devor and Wall, 1978).

## 3 Altered body representation following arm amputation

As highlighted above, the organising principles of the S1 somatotopy beyond nociception are highly ubiquitous. As such, S1 somatotopy provides an ideal model to address the question of brain remapping – can the properties of the map change, and in particular the boundaries between distinct body parts, once these have been established? Previous electrophysiology research in monkeys has identified extensive changes to the map features following amputation of a single digit (Merzenich et al., 1984), deafferentation of a nerve (Merzenich and Jenkins, 1993) or the entire arm (Pons et al., 1991). Here it was found that once neurons are deprived of their primary input, they become responsive to stimulation that activates the cortical neighbours of the deprived area. This activity change results in shifted boundaries of the body part map, termed cortical reorganisation. However, considering the key contribution of unmasking of already existing inputs in driving the shifted boundaries of the body map (Merzenich et al., 1983b), this description might be misleading. Perhaps the most dramatic demonstration of remapping occurs following arm deafferentation. Here the hand area of monkeys becomes responsive to inputs from the lower face – whose representation neighbours the hand area (though note that the activity in the deprived cortex is considerably smaller in amplitude to native face activity; see Figure 5 in (Kambi et al., 2014) for an example). Since hand and face inputs are segregated throughout the somatosensory hierarchy leading to S1, and since only sparse connections normally exist across the hand-face boundary (Chand and Jain, 2015), this process might reflect more profound changes to organising features of the body map.

**Fig. 2:**
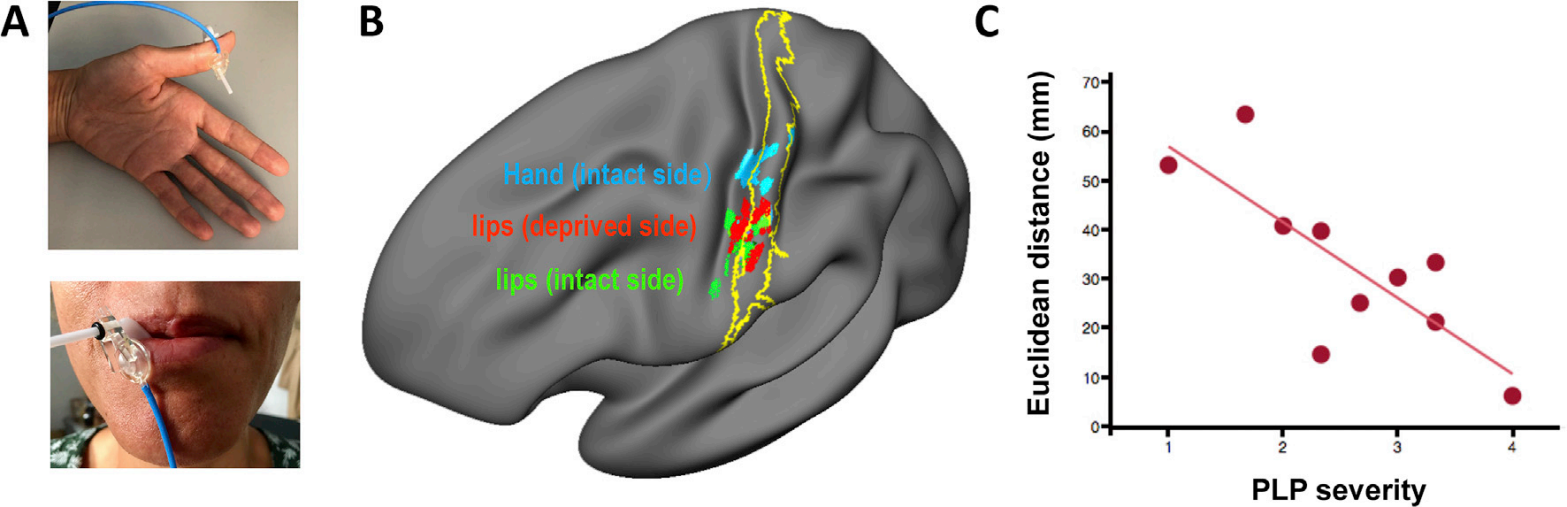
Shifted lip representation in the deprived cortex correlates with phantom limb pain (PLP). (A) Pictures illustrating the sensory stimulation applied over the thumb (top) and the lips (bottom). (B) fMRI activity during sensory stimulation applied over the intact thumb (blue), the lip on the deprived hemisphere (red) and the lip on the intact hemisphere (green) in amputees with PLP (n ​= ​10); projected to one hemisphere. The yelllow line shows a probabilistic deliniation of Broadmann areas 3b and 1 of S1. The stimulations were applied in different sessions in pseudorandomized order. The colored patches show the location of peak activity for the individual patients. The patches were sligthly enlarged (4 ​mm) for visualization purposes. The projections are carried out on a semi-inflated surface (all using surface-based analyses). C) Correlation between PLP severity (based on the Pain Intensity scale of the West Haven-Yale Multidimensional Pain Inventory adapted for PLP and Euclidean distances between the cortical representation of the thumb and the lips in the deafferented hemisphere (r ​= ​−0.79, p ​= ​0.006). These distances are measured between the lip representation (deafferented hemisphere) and intact thumb (with x-axis flipped to match the hemisphere of the lip). They are in mm and are calculated in the folded brain in standard space (i.e. standard brain). Based on HF’s unpublished data.

**Fig. 3:**
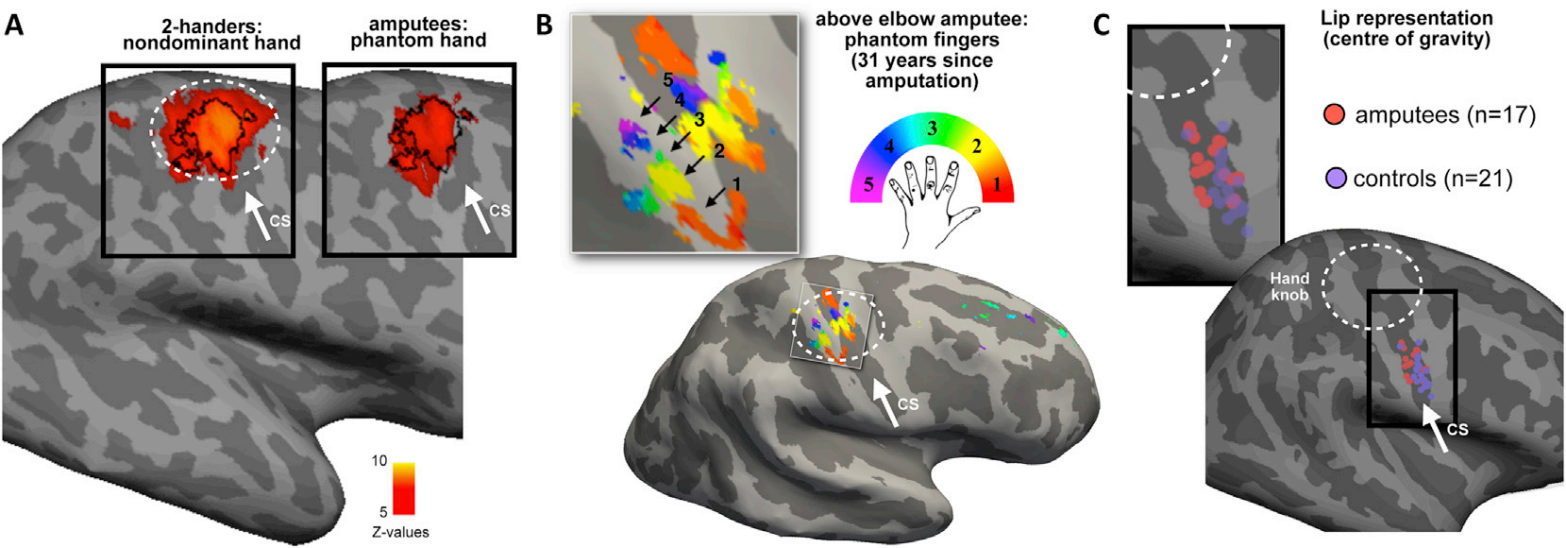
Persistent representation of the missing hand. (A) Activity group maps in controls (left) and amputees (right) during movements of the nondominant (controls) or phantom hand (amputees). White circle indicates the position of the anatomical hand knob. (B) A finger-selectivity map (using a travelling wave paradigm) for individual phantom finger movements reveals a complete hand somatotopy in primary somatosensory cortex of an amputee, with specific and adjacent clusters showing selectivity to specific phantom fingers. (C) Centre of gravity of lip activity clusters in individual participants (amputees, orange; controls, purple) reveals a medial shift in amputees’ lip representation, localised to the face area. On average, lips in the deprived hemisphere were shifted medially by 8 ​mm, compared to the intact hemisphere (note that the hand area is located 63 ​mm medially to the lips in controls). Images adapted from: (A) (Makin et al., 2013b); (B) (Kikkert et al., 2016); (C) (Makin et al., 2015b).

In humans, research characterising lower face representation in unilateral arm amputees has not identified clear facial activity in the missing hand cortex (Kikkert et al., 2018; Makin et al., 2013b) (Fig. 3C). Instead, multiple studies examining activity associated with stimulation of the lower face (both actively (Foell et al., 2014; Lotze et al., 2001; Makin et al., 2015b; Raffin et al., 2016) and passively (Elbert et al., 1994; Karl et al., 2001a; Yang et al., 1994) have found that the centre (or spatial extent) of the lip cluster becomes medially shifted in the deprived hemisphere, particularly with respect to the opposite (intact) hemisphere. Critically, the extent of this lip activity shift associates with PLP intensity (Flor et al., 1995; Karl et al., 2001; Fig. 2). PLP is a sub-class of phantom sensations that amputees report as arising from their missing limb (Henderson and Smyth, 1948). While these sensations range in their specific characteristics (spanning an extensive range of tactile, proprioceptive and kinesthetic sensations) they are often also experienced as bothersome and painful (Kooijman et al., 2000), usually developing into a chronic condition, which is difficult to treat (Weeks et al., 2010). This is due to the fact that we still do not have a complete understanding of its neural basis (Aternali and Katz, 2019). As such, the observed correlation between S1 remapping and PLP intensity has opened up new avenues for exploring the mechanisms behind, and novel treatments for, PLP.

In particular, it has been proposed that the displaced facial inputs caused by the deprivation-triggered remapping prompt aberrant processing in the S1 hand area, which may in turn be interpreted as phantom sensation or pain arising from the missing hand (Flor et al., 2006; Yang et al., 1994). As mentioned above, it was found that people who experience worse PLP also show a greater shift of their lower face representation towards the missing hand cortex (Flor et al., 1995). A similar association was never established for non-painful phantom sensations, which have been in turn linked with frontal and parietal activity (Andoh et al., 2017). The finding of a positive association between S1 remapping and PLP has been replicated using multiple paradigms and participant groups (Diers et al., 2010; Lotze et al., 1999; MacIver et al., 2008). It provided first evidence that reorganisation of the S1 map bears functional consequences, maladaptive at that, with new opportunities for PLP treatment. However, it also opens up a host of questions about the potential relationship between brain remapping and PLP, in particular – how would displaced input to S1 interact with other pain mechanisms to manifest the pain sensation? What is the time-course of cortical remapping with respect to PLP? Is this relationship between remapping and PLP also found following remapping of other body parts into the missing hand cortex? (see (Mezue and Makin, 2017) for further considerations). Some of these questions are starting to unravel by neuroimaging studies that focus on neural changes following various treatment approaches to PLP, as described below.

Human neuroimaging studies have also examined the neural representation of the missing hand itself. As mentioned above, phantom sensations can manifest in the form of kinaesthesia – the sense of movement that amputees experience when volitionally trying to move their phantom hand (Weeks et al., 2010). Previous research has demonstrated that when amputees are instructed to move their phantom hand, this results in motor output to the denervated motor neurons as evidenced with EMG (Reilly et al., 2006), demonstrating engagement of the motor system. fMRI studies taking advantage of this simple manipulation have shown that phantom movements activate both M1 and S1 ((Raffin et al., 2012); Fig. 3A), albeit these original studies could not reliably differentiate activity profiles of these two neighbouring regions. It has been demonstrated that activity elicited by phantom hand movements in the sensorimotor missing hand cortex positively correlates with PLP – people who experience more chronic PLP also exhibit greater activity when moving their phantom hands (Kikkert et al., 2018; Makin et al., 2013b). This finding highlights that mechanisms pertaining to persistent hand representation may also be relevant for PLP, involving either bottom up (peripheral (Vaso et al., 2014)) or top down processes (e.g., from the motor system (Kikkert et al., 2017)). These associations, however, still await a mechanistic framework by which phantom movements relate to PLP and it is therefore currently not clear whether there is a direct causal relationship between persistent representation and PLP (Kikkert et al., 2018), see further discussion below).

## 4 Insights on remapping and phantom pain from clinical studies

The research on S1 remapping after amputation has yielded novel interventions that target the presumed maladaptive brain remapping. In mirror treatment, the intact hand is moved while the patient views it in the mirror and perceives the mirror image as the phantom hand, while the phantom hand moves along (Chan et al., 2007) or while no phantom hand movement was present (Foell et al., 2014). Diers et al. (2010) observed that not all amputees activated the cortical representation of the phantom hand during observation of phantom movements through mirror training, and those that did, experienced less PLP. In a later study (Foell et al., 2014), it was found that successful reduction of PLP by several sessions of mirror treatment normalised lip activity, as evidenced by the degree of representational shift away from the missing hand area in comparison to the intact hemisphere. Normalisation of lip activity was found to significantly correlate with treatment success. Interestingly, patients with telescoping – where the phantom hand moves towards the residual limb – did not significantly benefit from mirror treatment. This was potentially due to the disjunct of the intact hand viewed in the mirror and the telescoped phantom percept, creating a perceptual mismatch. Augmented reality applications can overcome some limitations of mirror treatment (Barbin et al., 2016), and have shown that patients with a telescoped phantom can also benefit from mirror training, as demonstrated by treatment-related reorganisation (Thogersen et al., 2020). In addition, activity of a region in the inferior parietal cortex, which has been associated with body image maintenance, was predictive of reduced PLP. The latter data suggest that the non-painful perception of the phantom also modulates brain changes and PLP in amputees. A further study (De Nunzio et al., 2018) used a combination of phantom limb movement and sensory feedback in a small sample of amputees, and observed a trend towards a change in lip representation after treatment. Similar changes were reported for motor imagery (MacIver et al., 2008). These studies show that S1 changes and treatment-induced reductions in PLP covary, but the direction of the relationship, i.e. if the brain changes are a cause or consequence of the change in PLP cannot be determined from these studies. Studies that can assess causal relationships are needed.

Further studies using brain stimulation techniques, such as transcranial magnetic stimulation (TMS) and direct current stimulation (tDCS), can begin to address the question of causality by inducing cortical change and potential alterations to PLP. TMS studies (as reviewed in (Nardone et al., 2019)) show that the phantom limb representation in motor cortex is shifted in amputees with PLP and that the representation of neighbouring body parts enlarged, in line with findings from S1. A recent tDCS study (Kikkert et al., 2019) investigated the consequences of successful PLP reduction and preserved phantom hand representation after stimulation over the S1/M1 missing hand area, involving a functional task (phantom hand movements). PLP reduction was found to correlate with reduced phantom hand activity, in contrast to the findings on mirror treatment. However, this could be attributed to different activation paradigms (active phantom movement versus mirrored movements), as well as use-dependent changes related to the intact hand which is engaged in the mirror paradigms (as discussed below). Importantly, activity of the posterior insula and additional regions involved in pain processing emerged as an important mediator of pain relief and activity changes relating to phantom hand representation. These pain-related regions have not yet been as actively studied in amputees with PLP, compared to patients with other types of chronic pain. For example, research of chronic musculoskeletal pain has been focused on widespread brain alterations and less on S1 (Lopez-Sola et al., 2017). In this context, the above mentioned activity could be due to a greater association of PLP with changes in sensory rather than affective processing (Fuchs et al., 2018; Larbig et al., 2019).

Other research studying the neural correlates of PLP relief has focused on peripheral contributions to PLP. For example, targeted muscle and sensory reinnervation (TMR) of the amputated limb, which involves connecting nerves leading to the amputated limb to specific muscles that can drive a prosthetic limb has been shown to reduce PLP ((Dumanian et al., 2018); though see (Aternali and Katz, 2019)) and there is similar initial evidence for intraneural stimulation (Petrini et al., 2019). Finally, brain computer interfaces (BCI) have been explored as modulators of brain activity and PLP (Yanagisawa et al., 2016). BCI’s are promising avenues for treatment as they can provide new insights by up- and downregulating brain activity in the phantom cortex and related areas, whilst examining associated pain changes. Here it is worth considering more global consequences of neuromodulation on somatosensation. To date, the relationship between amputation-triggered changes to S1 and altered somatosensation is far from clear, since no systematic studies exist that have related various sensory modalities to brain changes. Studies on psychophysical properties have found both enhanced and deficient sensory processing (Hunter et al., 2005; Li et al., 2015).

## 5 Additional contributors to remapping beyond deprivation

As mentioned above, remapping following amputation has been considered to be primarily triggered by the loss of sensory input, driving unmasking of weak or silent inputs (Merzenich et al., 1983b), and potentially promoting long-term potentiation mechanisms (Polley et al., 1999). According to this account, inputs that already have access to the deprived cortex, such as topographic neighbours, are more likely to be expressed in it following deprivation, resulting in a shift or expansion of an existing boundary. However, recent evidence in neuroimaging demonstrates violations of this rationale. For example, it was found that the residual arm, which is immediately neighbouring the missing hand cortex, does not remap into the deprived sensorimotor cortex in amputees, relative to controls (Makin et al., 2013a). Converseley, multiple studies have documented increased activity in the sensorimotor missing hand cortex for the intact hand (Bogdanov et al., 2012; Makin et al., 2013a; Philip and Frey, 2014; Raffin et al., 2016; Wesselink et al., 2019). Considering that the deprived cortex is normally inhibited by the intact hand cortex, it is difficult to resolve this finding with classical interhemispheric inhibition mechanisms (e.g. as suggested for stroke (Ward and Cohen, 2004)). Instead, it has been proposed that the remapping of the intact hand into the missing hand hemisphere relates to compensatory usage (Makin et al., 2013a).

It has long been observed that the receptive field (RF) properties of the remapped cortex gradually change over a long time-scale (Merzenich et al., 1983b). It has been suggested that the initial remapping triggered by deprivation will become refined by inputs due to daily hand usage involving compensatory behaviours (Churchill et al., 1998; Elbert et al., 1997). Since altered input is known to drive and shape brain organisation (Recanzone et al., 1992; Wang et al., 1995), it is reasonable to expect that adapted behaviour substituting for the missing hand function can shape remapping. This is akin to recent studies in individuals with congenital hand loss, who exhibit remapping of multiple body parts, which are used for compensatory behaviours, including the feet, residual arm and lips (Hahamy et al., 2017; Hahamy and Makin, 2019; Stoeckel et al., 2009) into the missing hand cortex. Considering that deprived cortex has been demonstrated to undergo network-level reorganisation in functional connectivity (Makin et al., 2015a), and that the connectome of the deprived cortex is wired to support hand function (Glasser et al., 2016; Graziano and Aflalo, 2007), inputs relating to typical hand function (e.g. manipulating objects) might consolidate more favourably. While still awaiting causal validation for a relationship between changed habitual behaviour and brain remapping in humans with congenital or acquired amputation (Dempsey-Jones et al., 2019), this potential process sheds new light on the classical findings. For example, consider the original studies in monkeys showing remapping of the mouth into the hand cortex. If these monkeys tended to use their mouth to substitute for their injured hand function, this could have contributed to the resulting remapping of the mouth into the hand area. It also provides new context to the aforementioned mirror-treatment studies which used intact hand movements to probe representations in the missing hand cortex (Diers et al., 2010). Currently, there is no systematic evidence that intact hand remapping associates with PLP, though few studies explored this potential link (Makin et al., 2013a; Philip and Frey, 2014; Raffin et al., 2016).

Prosthetic limb usage was also demonstrated to associate with cortical remapping and PLP (Lotze et al., 1999), but here again causality needs to be inferred with caution. It is known that individuals suffering from PLP are less likely to wear a prosthetic limb due to discomfort (Jang et al., 2011). It has also been demonstrated that individuals that use their prosthetic limb less (or more generally their residual arm (Makin et al., 2013a)), tend to use their intact hand more, presumably due to the need for alternative compensatory strategies. Any of these factors could potentially directly impact S1 organisation and beyond (van den Heiligenberg et al., 2018), or serve as an indirect moderating factor for a relationship between S1 remapping, PLP and persistent representation.

**Fig. 4:**
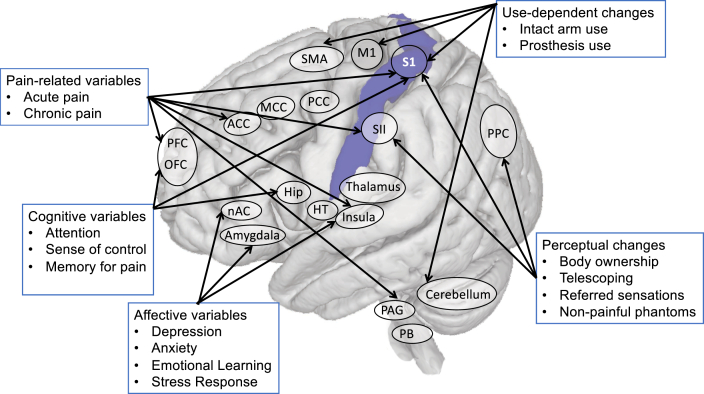
Multiple drivers of remapping in primary somatosensory cortex and phantom limb pain (PLP). These variables have been shown to modulate either S1 organisation, PLP, or both. These multiple factors, and any interactions between them, need to be considered in addition to injury-related changes in any future mechanistic analyses of S1 remapping and PLP. Abbreviations. ACC, anterior cingulate cortex; MCC, midcingulate cortex; PCC, posterior cingulate cortex; Nac, Nucleus accumbens; BG, basal ganglia; PAG, periaqueductal grey; PFC, prefrontal cortex; OFC, orbitofrontal cortex; M1, primary motor cortex; S1, primary somatosensory cortex; SMA, supplementary motor area; SII, secondary somatosensory cortex; PB, parabrachial nucleus; PPC, posterior parietal cortex; Hip, hippocampus; HT, hypothalamus.

As already noted in section 4 on clinical studies, contextual variables also influence S1 remapping. For example, telescoping, which is associated with PLP and greater S1 remapping, was related to less PLP reduction and less changes in the mouth to hand cortical distance following mirror treatment (Foell et al., 2014). Other evidence for the instability of the S1 finger maps provide further considerations, which could impact the evidence for remapping observed in amputees. For example, acute pain (which will differ across amputees during the scanning session) has been shown to increase cortical distances in S1 (Buchner et al., 2000). Furthermore, Braun and colleagues (Braun et al., 2000, 2002) observed that the hand map can change depending on task requirements and attentional focus. These results confirm earlier suggestions that the fine features of the body maps in S1 are under-determined to an extent, and as such, could dynamically adapt to internal and external conditions within short time periods. Consequently, the context of the experimental paradigm and the details of the analysis could massively impact the boundaries of the body map being described by the researcher. This does not preclude high stability of finger maps under similar conditions (Kolasinski et al., 2016). Fig. 4 depicts some of the factors that can impact on remapping in S1.

## 6 Probing reorganisation using multivariate analysis techniques

Recent multivoxel analysis techniques offer a new lens through which somatotopic (re)mapping can be characterised by providing us with the means to quantify selectivity in terms of how distinct the representation of one body part is to another (for example, linear classifiers or the Mahalanobis distances observed between two representations in a given brain area). Moreover, these techniques provide the means to consider the type and extent of available information underlying the body map. The simple assumption here is that a brain area representing a given body part should contain distinct information about the functional features most relevant to the representation of that body part. For example, in S1 there are distinct neural representations of specific frequencies of tactile stimulus properties (Bensmaia, 2008), which might not be easily separable based on net activity levels, but result in distinct multivariate representational motifs (Kim et al., 2016). Representational similarity analysis (RSA) permits the consideration of functional attributes beyond selectivity of response. For example, it allows characterisation of inter-digit similarity or overlap (Diedrichsen and Kriegeskorte, 2017). Using this approach, it has been elegantly demonstrated that the canonical inter-digit representational structure in the S1 hand area can be best described in terms of daily hand use: those digits that form kinematic synergies to afford our daily interactions with objects and tools also show greater similarity in their representation (Ejaz et al., 2015).

7T fMRI and multivariate analysis approaches have been recently used to investigate S1 hand representation in amputees. With respect to persistent hand representation, 7T fMRI allowed us to uncover S1 digit maps, even up to three decades after amputation ((Kikkert et al., 2016); Fig. 3B). A complete digit map was also found in the missing hand cortex of an amputee with a brachial plexus avulsion (causing peripheral deafferentation), and was abolished when the participant was asked to imagine moving the phantom hand, rather than attempting to move it. As such, it appears that these maps reflect inputs from the motor system which are somatotopically structured. Multivoxel analysis techniques allow us to confirm that the representation of the missing hand, as probed using phantom hand movements (or at least attempted movements), shares the same representational features as a normal hand. In particular, we recently found that the unique configuration of inter-finger representational structure (previously linked with everyday experience), is entirely preserved after an average of 18 years since amputation (Wesselink et al., 2019). We found that across participants there was a significant relationship between the typicality of phantom hand representation and the sense of phantom kinaesthesia, specifically the number of digits that amputees perceived to be moving during the task. However, we found no significant correlations with either acute or chronic PLP, or the vividness of non-painful phantom sensations (as revealed in a linear regression model). In fact, typical hand representation was also found in the few amputees who did not experience any phantom sensations, suggesting it is a canonical organising principle, rather than a neural correlate of the phantom hand. A further study by Bruurmijn and colleagues, who used brain decoding to separate representations of multiple phantom hand gestures, was able to successfully decode the different gestures well above chance (Bruurmijn et al., 2017). Although decoding accuracy was reduced when compared with controls in the earlier sub-divisions of S1, this group difference was resolved in the higher sub-divisions (BA 1 and 2), indicating that the preserved representation of the phantom hand is at least partially maintained by higher order processing. Here again, no significant correlations were found with PLP (though considering the small sample size of n ​= ​8, this null result should be regarded with caution).

Similar techniques have also been used to study the intact hand, specifically the increased ipsilateral activity that has been previously observed in the missing hand cortex. Previous research in two-handed adults demonstrated that the ipsilateral hand area contains finger-specific information which mirrors the contralateral representation of the same finger (Diedrichsen et al., 2013; Diedrichsen et al., 2018). This approach allows us to ask whether information content is greater for the ipsilateral representation of amputees’ intact hand relative to controls. In a recent study we found that ipsilateral inter-finger dissimilarity, underlying digit selectivity, was not significantly different between amputees and controls. This result indicates that the increased intact hand activity reported in amputees might not underlie increased functional processing (Wesselink et al., 2019). Similar evidence from stroke (Ejaz et al., 2018) and focal dystonia patients (Ejaz et al., 2016), also showed no changes in inter-finger representational features using RSA. This is in contrast to previous studies demonstrating that hand somatotopy is altered in these groups of interest (Flor and Diers, 2009), as mentioned in our introduction. Since this recent evidence demonstrates that information processing of inter-finger representation is unchanged, it is therefore possible that the previously documented map changes, which were based on measures of net activity changes, do not entail reorganisation of representations.

## 7 Concluding remarks

Our review shows that there is no simple relationship between somatosensory map reorganisation, PLP and preserved hand representation. This perspective converges with recent studies in related clinical conditions, for example complex regional pain syndrome (Mancini et al., 2019) and peripheral neuropathy (Maeda et al., 2014), showing that the previously assumed relationship between S1 remapping and pain requires further consideration. Although injury-related plasticity mechanisms may be the original driver of map changes, over time a number of additional factors such as use of a prosthesis, intact hand use, alterations in body representation as a consequence of amputation and processing in other regions, particularly with regards to pain processing, may interact with injury-related remapping. Furthermore, the close and bidirectional connections of somatosensory and motor cortex are also likely to impact S1 organisation. These interactions need to be examined and related to functional changes. Thirdly, the traditional focus on S1 remapping due to changed selectivity to particular body parts is probably incomplete, considering net activity changes might not adequetly reveal the underlying functional processing. Moreover, related research in monkeys (Kambi et al., 2014) and humans (Hahamy et al., 2019) indicate that sensorimotor cortical remapping might reflect changes in sub-cortical terminals (e.g. brainstem, basal ganglia) or cerebellum. Future research should aim to study PLP within this broader context, while taking into consideration the role of multiple brain networks and other contextual factors that can alter or stabilise the S1 body map at muliple time scales.

## CRediT authorship contribution statement

**Tamar R. Makin:** Conceptualization, Writing - original draft. **Herta Flor:** Conceptualization, Writing - original draft.
